# Cross-sectional associations of plasma vitamin D with cerebral β-amyloid in older adults at risk of dementia

**DOI:** 10.1186/s13195-018-0371-1

**Published:** 2018-04-25

**Authors:** Fati Nourhashemi, Claudie Hooper, Christelle Cantet, Catherine Féart, Isabelle Gennero, Pierre Payoux, Anne Sophie Salabert, Sophie Guyonnet, Philipe De Souto Barreto, Bruno Vellas, for the Multidomain Alzheimer Preventive Trial/Data sharing Alzheimer (DSA) study group, Bruno Vellas, Sophie Guyonnet, Isabelle Carrié, Lauréane Brigitte, Catherine Faisant, Françoise Lala, Julien Delrieu, Hélène Villars, Emeline Combrouze, Carole Badufle, Audrey Zueras, Sandrine Andrieu, Christelle Cantet, Christophe Morin, Gabor Abellan Van Kan, Charlotte Dupuy, Yves Rolland, Céline Caillaud, Pierre-Jean Ousset, Françoise Lala, Bertrand Fougère, Sherry Willis, Sylvie Belleville, Brigitte Gilbert, Francine Fontaine, Jean-François Dartigues, Isabelle Marcet, Fleur Delva, Alexandra Foubert, Sandrine Cerda, Marie-Noëlle-Cuffi, Corinne Costes, Olivier Rouaud, Patrick Manckoundia, Valérie Quipourt, Sophie Marilier, Evelyne Franon, Lawrence Bories, Marie-Laure Pader, Marie-France Basset, Bruno Lapoujade, Valérie Faure, Michael Li Yung Tong, Christine Malick-Loiseau, Evelyne Cazaban-Campistron, Françoise Desclaux, Colette Blatge, Thierry Dantoine, Cécile Laubarie-Mouret, Isabelle Saulnier, Jean-Pierre Clément, Marie-Agnès Picat, Laurence Bernard-Bourzeix, Stéphanie Willebois, Iléana Désormais, Noëlle Cardinaud, Marc Bonnefoy, Pierre Livet, Pascale Rebaudet, Claire Gédéon, Catherine Burdet, Flavien Terracol, Alain Pesce, Stéphanie Roth, Sylvie Chaillou, Sandrine Louchart, Kristelle Sudres, Nicolas Lebrun, Nadège Barro-Belaygues, Jacques Touchon, Karim Bennys, Audrey Gabelle, Aurélia Romano, Lynda Touati, Cécilia Marelli, Cécile Pays, Philippe Robert, Franck Le Duff, Claire Gervais, Sébastien Gonfrier, Yannick Gasnier, Serge Bordes, Danièle Begorre, Christian Carpuat, Khaled Khales, Jean-François Lefebvre, Samira Misbah El Idrissi, Pierre Skolil, Jean-Pierre Salles, Carole Dufouil, Stéphane Lehéricy, Marie Chupin, Jean-François Mangin, Ali Bouhayia, Michèle Allard, Frédéric Ricolfi, Dominique Dubois, Marie Paule Bonceour Martel, François Cotton, Alain Bonafé, Stéphane Chanalet, Françoise Hugon, Fabrice Bonneville, Christophe Cognard, François Chollet, Pierre Payoux, Thierry Voisin, Julien Delrieu, Sophie Peiffer, Anne Hitzel, Michèle Allard, Michel Zanca, Jacques Monteil, Jacques Darcourt, Laurent Molinier, Hélène Derumeaux, Nadège Costa, Christian Vincent, Bertrand Perret, Claire Vinel, Pascale Olivier-Abbal, Sandrine Andrieu, Christelle Cantet, Nicola Coley

**Affiliations:** 1 Gérontopôle, Department of Geriatrics, Toulouse University Hospital, Toulouse, France; 2 INSERM UMR 1027, Toulouse, France; 3 University of Toulouse III, Toulouse, France; 4 Department of Epidemiology and Public Health, CHU Toulouse, Toulouse, France; 5 University of Bordeaux, Inserm, Bordeaux Population Health Research Center, Team LEHA, UMR 1219, F-33000 Bordeaux, France; 6 UMR1043 Centre de Physiopathologie Toulouse Purpan, Université de Toulouse, UPS, INSERM, Toulouse, France; 7 Institut Federatif de Biologie, CHU Toulouse, Purpan University Hospital, Toulouse, France; 8 UMR 1214, Toulouse Neuroimaging Center, University of Toulouse III, Toulouse, France; 9 Department of Nuclear Medicine, Toulouse University Hospital, Toulouse, France

**Keywords:** Vitamin D, Beta-amyloid, Alzheimer’s disease, Positron emission tomography

## Abstract

**Background:**

Vitamin D deficiency is associated with an increased risk of Alzheimer’s disease and increased beta-amyloid (Aβ) in animals. Hence we sought to investigate the relationship between plasma 25-hydroxyvitamin D (25(OH)D) and cerebral Aβ in older adults with subjective memory complaints.

**Methods:**

This is a secondary analysis of the Multidomain Alzheimer Preventive Trial. Participants were 178 dementia-free individuals aged 70 years or older with data on plasma 25(OH)D and cerebral Aβ load assessed by [^18^F]-florbetapir positron emission tomography. Plasma 25(OH)D was measured at study baseline using a commercially available electro-chemiluminescence competitive binding assay. Standard uptake value ratios (SUVRs) were generated using the cerebellum as a reference. Brain regions assessed included the cortex, anterior cingulate, anterior putamen, caudate, hippocampus, medial orbitofrontal cortex, occipital cortex, parietal cortex, pons, posterior cingulate, posterior putamen, precuneus, semioval centre and temporal cortex. Associations were explored using fully adjusted multiple linear regression models.

**Results:**

Participants had a mean (SD) age of 76.2 years (4.4) and 59.6% were female. The mean (SD) plasma 25(OH)D level was 22.4 ng/ml (10.8) and the mean (SD) cortical SUVR was 1.2 (0.2). We did not find any cross-sectional associations (*p* > 0.05) between baseline 25(OH)D levels and Aβ in any of the brain regions studied.

**Conclusions:**

These preliminary results suggest that circulating 25(OH)D is not associated with cerebral Aβ in older adults. Further longitudinal studies with the measurement of mid-life vitamin D status are required to explore the relationship between vitamin D and Aβ accrual over time, thereby circumventing the shortfalls of a cross-sectional study.

## Background

Vitamin D is a fat-soluble steroid hormone that seems crucial for brain health in humans [1–5]. Low vitamin D status is common amongst the elderly and is considered a major health problem [6–8]. Studies have shown that vitamin D insufficiency is associated with a higher risk of Alzheimer’s disease (AD) [9–16] and accelerated cognitive decline [12, 17, 18], although some conflicting results are reported [19–21]. Randomized controlled trials (RCTs) investigating the effects of vitamin D supplementation (alone or in combination with other drugs) on cognitive decline and AD onset are limited [22–25] and have largely proven unsuccessful to date [22, 23, 25].

Despite the controversy surrounding the role of vitamin D in cognitive function and AD, animal studies suggest that vitamin D hypovitaminosis results in increased brain beta-amyloid (Aβ) [26] and vitamin D promotes a reduction in Aβ brain burden [27, 28]. In human studies, vitamin D has been shown to increase plasma Aβ, particularly in older adults, suggestive of decreased brain Aβ [29]. Furthermore, vitamin D status assessed through a dietary questionnaire has been associated with less Aβ in AD-vulnerable brain regions in subjects at risk of AD [30, 31]. However, to the best of our knowledge, studies have not addressed the relationship between circulating vitamin D (measured biochemically) and cerebral Aβ. Thus, we examined the cross-sectional associations between 25-hydroxyvitamin D (25(OH)D), the major circulating form of vitamin D, and cerebral Aβ in older adults at risk of dementia. We hypothesized that 25(OH)D would be inversely associated with Aβ.

## Methods

### The Multidomain Alzheimer Preventive Trial, ethics and approval

Data were obtained from a [^18^F]-florbetapir positron emission tomography (PET) study carried out as an ancillary project to the Multidomain Alzheimer Preventive Trial (MAPT) [32] (ClinicalTrials.gov, NCT00672685), a large multicentre, phase III, randomized, placebo-controlled trial. The MAPT was designed to assess the effects of omega 3 polyunsaturated fatty acid (n-3 PUFA) supplementation alone or in combination with a multidomain intervention (involving nutritional and exercise counselling and cognitive training), compared to placebo, in slowing cognitive decline in older adults with subjective memory complaints (*n* = 1680). Both the MAPT and the ancillary [^18^F]-florbetapir PET study were approved by the ethics committee in Toulouse (CPP SOOM II) and written consent was obtained from all participants.

### Participants

At inclusion, subjects were community-dwelling men and women without dementia, aged ≥ 70 years, who met at least one of the following criteria: spontaneous memory complaints, limitation in executing ≥ 1 Instrumental Activity of Daily Living, or slow gait speed (≤ 0.8 m/s). Participants of this study were 180 individuals who had data on plasma 25(OH)D and cerebral Aβ load. However, two participants were excluded from the present analysis because they had CDR ≥ 1 (suggestive of dementia) at the clinical visit closest to PET-scan assessment. Thus, a total of 178 participants were included in the study described here.

### [^**18**^F]-Florbetapir positron emission tomography

PET scans as a measure of cerebral Aβ load were performed using [^18^F]-florbetapir as described previously [32, 33]. PET data acquisitions commenced 50 min after injection of a mean of 4 MBq/kg weight of [^18^F]-Florbetapir. Radiochemical purity of [^18^F]-Florbetapir was superior to 99.5%. Regional standard uptake value ratios (SUVRs) were generated from semi-automated quantitative analysis with the whole cerebellum used as the reference region. Cortical-to-cerebellar SUVRs (cortical-SUVRs) were obtained using the mean signal of the predefined cortical regions: frontal, temporal, parietal, precuneus, anterior cingulate and posterior cingulate as described previously [34]. Other brain regions independently assessed were the anterior cingulate, anterior putamen, caudate, hippocampus, medial orbitofrontal cortex, occipital cortex, parietal cortex, pons, posterior cingulate, posterior putamen, precuneus, semioval centre and temporal cortex. A Quality Control procedure was carried out using a semi-quantification-based method. PET scans were performed an average of 18.1 ± 8.8 months after baseline.

### Measurement of 25(OH)D

Total plasma 25(OH)D (D3 and D2 forms) was measured using a commercially available electro-chemiluminescence competitive binding assay (Cobas, Roche) in baseline plasma samples. In brief, plasma (15 μl) was denatured to release bound 25(OH)D from vitamin D binding protein (VDBP). The sample was then incubated with recombinant ruthenium-labelled VDBP to enable complex formation. Biotinylated 25(OH)D was subsequently added and the entire complex became bound to the electrode through an interaction of biotin with streptavidin-coated micro-particles, which are captured on the electrode surface. Unbound substance was subsequently removed and a voltage was applied to the electrode which induced a chemiluminescent emission that was quantified using a Roche Cobas 8000 e602 analyser (Roche Diagnostics, Mannheim, Germany). The concentrations of 25(OH)D (ng/ml) were determined against a standard curve.

### Confounding variables

We selected the following confounding variables on the basis of data availability and the literature on AD and vitamin D [35, 36]: age at PET-scan assessment, gender, body mass index (BMI), season of blood collection (four categories: autumn, winter, spring, summer), educational level, cognitive status assessed at the clinical visit closest to the PET scan (Mini Mental State Examination (MMSE): score out of 30), time interval (months) between baseline and the PET scan, MAPT group allocation (four groups: placebo, multidomain intervention, n-3 PUFA supplementation, multidomain intervention + n-3 PUFA supplementation) and apolipoprotein E ε4 (ApoE ε4) genotype (carriers of at least one ε4 allele versus non-carriers).

### Statistical analysis

Descriptive characteristics are presented as mean ± standard deviation (SD) or absolute values/percentages. The relationship between plasma 25(OH)D and cerebral Aβ load was not an a-priori hypothesis of the MAPT. After completing the analysis of the primary hypotheses in the MAPT, we performed additional post-hoc analyses using multiple linear regression models to evaluate the cross-sectional relationships between plasma 25(OH)D and cerebral brain Aβ load measured in SUVRs (both as continuous variables) adjusting for all confounders. We ran three separate sensitivity analyses in order to substantiate our main analysis exploring the relationship between 25(OH)D and cortical Aβ. Firstly, we classified the 25(OH)D data according to the cut-off values < 20 ng/ml, ≥ 20 but ≤ 30 ng/ml and > 30 ng/ml which are defined as 25(OH)D deficiency, insufficiency and sufficiency [37]. The cut-off value of < 10 ng/ml, which has been associated with worse cognitive outcomes, was not used since there were only 16 out of 178 subjects (9%) with 25(OH)D in this range. The class containing values of 25(OH)D >  30 ng/ml was used as the reference. We then ran multiple linear regression analysis adjusted for all confounders. Secondly, we dichotomized the dependent variable, cortical Aβ load, with a positivity threshold of mean cortical SUVR ≥ 1.17 [33, 38] and then performed logistic regression adjusted for all confounders. Thirdly, we used de-seasonalized 25(OH)D values calculated according to the periodic function *y*_*t*_ = *B*_0_ + *B*_1_ sin(2π*t*/365) + *B*_2_ cos(2π*t*/365) [12, 39] to assess the relationship between 25(OH)D and cortical Aβ (both as continuous variables) using multiple linear regression analysis adjusted for all confounders (minus season categorized into four classes). To explore the role of apolipoprotein E (ApoE) ε4 genotype, the major risk factor for sporadic SD [40], on the association between 25(OH)D and cortical Aβ (both as continuous variables) we ran a linear regression analysis with the introduction of an interaction term between ApoE ε4 genotype and 25(OH)D. There was no correction for multiple comparisons: *p* < 0.05 was considered statistically significant. Statistical analyses were performed using SAS software version 9.4 (SAS Institute Inc., Cary, NC, USA).

## Results

### Sample characteristics

Clinical and demographic characteristics of the study participants are presented in Table 1. The mean age of the participants was around 76 years and approximately 60% of the subjects were female. Participants exhibited a high educational level and just less than half had a CDR score of 0.5. Approximately one-quarter of the subjects were ApoE ε4 carriers. The mean (SD) plasma 25(OH)D level was 22.4 ng/ml (10.8) and the mean (SD) cortical SUVR was 1.2 (0.2).

**Table 1:** Participant characteristics

Variable	Value (*n* = 178)
Age (years)	76.2 ± 4.4
Sex, women	106 (59.6%)
Education	
No diploma or primary school certificate	47 (26.6%)
Secondary education no high-school diploma	49 (27.7%)
High school diploma	24 (13.6%)
University level	57 (32.2%)
MAPT group allocation	
Multidomain intervention	39 (21.9%)
n-3 PUFA supplementation	42 (23.6%)
Multidomain intervention and n-3 PUFA supplementation	49 (27.5%)
Placebo	48 (27.0%)
Season of vitamin D measurement	
Winter	71 (39.9%)
Spring	37 (20.8%)
Summer	15 (8.4%)
Autumn	55 (30.9%)
BMI (kg/m^2^)	26.6 ± 4.0
CDR 0.5	79 (44.4%)
MMSE score (/30)	28.3 ± 1.6
ApoE ε4 carriers^a^	42 (25.6%)
25-Hydroxyvitamin D (ng/ml)	22.4 ± 10.8
25-Hydroxyvitamin D < 20 ng/ml	81 (45.5%)
25-Hydroxyvitamin D ≥ 20 ng/ml ≤ 30 ng/ml	56 (31.5%)
25-Hydroxyvitamin D > 30 ng/ml	41 (23.0%)
Cortical SUVR	1.2 ± 0.2
Cortical SUVR positive (≥ 1.17)	75 (42.1%)

Age, CDR, BMI and MMSE score closest to the PET scan are presented

Data expressed as mean ± standard deviation or absolute value (percentage)

*BMI* body mass index, *CDR* Clinical Dementia Rating, *MAPT* Multidomain Alzheimer Preventive Trial, *n-3 PUFA* omega-3 polyunsaturated fatty acid, *MMSE* Mini Mental State Examination, *ApoE* apolipoprotein E, *SUVR* standard uptake ratio values

^a^ApoE status available for *n* = 164

### Exploration of the relationship between 25(OH)D and cerebral Aβ

Neither cortical Aβ load nor Aβ levels present in the anterior cingulate, anterior putamen, caudate, hippocampus, medial orbitofrontal cortex, occipital cortex, parietal cortex, pons, posterior cingulate, posterior putamen, precuneus, semioval centre and temporal cortex were associated with plasma 25(OH)D after adjustment for all confounders (Table 2).

**Table 2:** Exploration of the association of 25(OH)D with cerebral Aβ

Brain region	Unadjusted model			Adjusted model		
	*B* coefficient	SE	*p*	*B* coefficient	SE	*p*
Cortex	− 0.001	0.001	0.587	− 0.001	0.001	0.376
Anterior cingulate	−0.001	0.002	0.378	−0.002	0.002	0.325
Anterior putamen	0.000	0.001	0.972	−0.000	0.001	0.880
Caudate	−0.001	0.001	0.502	−0.000	0.001	0.872
Hippocampus	0.000	0.001	0.840	0.001	0.001	0.495
Medial orbitofrontal cortex	−0.000	0.001	0.928	−0.000	0.001	0.794
Occipital cortex	−0.001	0.001	0.351	−0.001	0.001	0.291
Parietal cortex	−0.000	0.001	0.930	−0.001	0.001	0.662
Pons	0.000	0.001	0.941	0.000	0.001	0.698
Posterior cingulate	−0.000	0.001	0.779	−0.001	0.001	0.461
Posterior putamen	0.000	0.001	0.623	0.000	0.001	0.822
Precuneus	−0.001	0.002	0.471	−0.002	0.002	0.287
Temporal cortex	−0.001	0.001	0.479	−0.001	0.001	0.240
Semioval centre	−0.001	0.001	0.646	0.000	0.001	0.803

The unadjusted linear regression model in the main analysis included all 178 participants who underwent [^18^ F]-florbetapir positron emission tomography imaging. Cortical-to-cerebellar standard uptake value ratios were obtained using the mean signal of the following predefined cortical regions: frontal, temporal, parietal, precuneus, anterior cingulate and posterior cingulate. Other brain regions were independently assessed for Aβ in relation to the cerebellum as a reference region. The adjusted model contained fewer subjects (*n* = 176) due to missing data on confounders

*Aβ* beta-amyloid, *25(OH)D* 25-hydroxyvitamin D, *SE* standard error

### Sensitivity analysis

Categorization of the 25(OH)D data and dichotomization of the Aβ data showed comparable results to the main analysis using cortical Aβ (Table 3). Exploring the relationship between de-seasonalized vitamin D values and cortical Aβ load also corroborated the main analysis using cortical Aβ (Table 3).

**Table 3:** Sensitivity analyses exploring the association of 25(OH)D with cerebral Aβ

	Unadjusted model			Adjusted model		
	*B* coefficient or odds ratio*	SE or 95% CI*	*p*	*B* coefficient or odds ratio*	SE or 95% CI*	*p*
Sensitivity analysis A: 25(OH)D in classes^a^						
25(OH)D < 20 ng/ml	0.005	0.033	0.869	0.010	0.034	0.775
25(OH)D ≥ 20 ng/ml ≤ 30 ng/ml	0.061	0.036	0.089	0.039	0.035	0.273
Sensitivity analysis B: logistic regression^b^						
25(OH)D < 20 ng/ml	1.021*	0.477,2.188*	0.957	1.038*	0.421,2.557*	0.936
25(OH)D ≥ 20 ng/ml ≤ 30 ng/ml	1.059*	0.468,2.395*	0.891	0.769*	0.299,1.978*	0.585
Sensitivity analysis C: de-seasonalized 25(OH)D (continuous)^c^	−0.001	0.001	0.600	−0.001	0.001	0.421

^a^Multiple linear regression was run to explore the associations between cortical Aβ and 25(OH)D classified according to the cut-off values < 20 ng/ml, ≥ 20 but ≤ 30 ng/ml and > 30 ng/ml adjusting for all confounders

^b^Logistic regression was performed with Aβ dichotomized with a positivity threshold of mean cortical standard uptake value ratio ≥ 1.17 and 25(OH)D in classes with adjustment for all confounders

^c^De-seasonalized 25(OH)D values were used to assess the relationship between cortical Aβ and 25(OH)D both as continuous variables using a multiple linear regression model adjusted for all confounders. The adjusted models contained fewer subjects (*n* = 176) due to missing data on confounders

*Aβ* beta-amyloid, *CI* confidence interval, *SE* standard error, *25(OH)D* 25-hydroxyvitamin D

### Exploratory analysis

Next we explored the relationship between 25(OH)D and cortical Aβ as a function of ApoE ε4 status. However, the interaction between 25(OH)D and ApoE ε4 genotype was not associated with cortical Aβ load (*p* = 0.434).

## Discussion

We have shown that vitamin D measured as 25(OH)D was not associated with cerebral Aβ independently or as a function of ApoE ε4 status. To the best of our knowledge, this is the first study to explore the associations between circulating plasma vitamin D (measured biochemically) and cerebral Aβ.

Our findings were against our original hypothesis based on animal experiments [27, 28] and human studies using nutritional questionnaires to assess the associations between vitamin D status and cerebral Aβ [30, 31]. However, the participants of these human studies were younger and cognitively normal. It might also be that dietary questionnaires do not best capture vitamin D status especially in those with memory complaints. Moreover, sun exposure is the major source of vitamin D.

Our findings are in line with those showing that vitamin D concentrations are not associated with cognitive decline or AD [19–21], although a number of reports to the contrary are published [9–18]. Considering that RCTs investigating the effects of vitamin D supplementation on brain health are negative to date [22, 23, 25], it could be hypothesized that observational studies reporting a significant association between vitamin D status and cognitive decline or AD risk might be prone to reverse causality bias. Alternatively, the failure of vitamin D trials might be attributed to the short duration of therapy or the sub-optimal timing of vitamin D supplementation. Timing has recently been highlighted as an important criterion in the relationship between vitamin D and cognition [18, 41]. Indeed, vitamin D status mid-life has been associated with cognition 10 years later [42]. Thus, the duration and time window of vitamin D hypovitaminosis might dictate pathological changes associated with AD in older age. In the same vein, the assessment of cerebral Aβ in later life does not provide information on the slow pathophysiological accrual of Aβ [43] and the role of vitamin D. Perhaps as with cognition, mid-life vitamin D deficiency might contribute more to Aβ deposition than vitamin D deficiency in later life. Thus, in this study we might have failed to detect an association between vitamin D status and cerebral Aβ load due to the inappropriate timing of measurements; however, plasma samples in mid-life were not available in the MAPT to test further hypotheses.

It is also feasible that vitamin D hypovitaminosis is linked to cognitive decline via Aβ-independent processes. Indeed, 25(OH)D insufficiency is associated with an increase in white matter abnormalities indicative of cerebrovascular disease [44, 45] and decreasing 25(OH)D plasma levels are associated with an increased risk of ischaemic stroke [46]. Thus, it is plausible that vitamin D might be associated with dementia of a more vascular nature. Vitamin D has also been shown to regulate the synthesis of neurotrophins [47, 48] and therefore vitamin D hypovitaminosis could potentially promote neuronal death and cognitive decline. Moreover, vitamin D regulates the expression of a number of neurotransmitters including acetylcholine [49] and dopamine [50] and the expression of the enzyme involved in the rate-limiting step of catecholamine synthesis [51], which in turn might impact cognition. In addition, vitamin D inhibits the synthesis of inducible nitric oxide synthase [52], downregulates reactive oxygen species [53], upregulates the antioxidant glutathione [54] and inhibits the expression of pro-inflammatory cytokines [55]. Thus, vitamin D hypovitaminosis might potentiate inflammation and oxidative damage to neurons, hence promoting cognitive deterioration.

Exploratory analysis showed that there was no interaction between 25(OH)D and ApoE ε4 genotype to modify cortical Aβ. It has been shown previously that ApoE ε4 carriers have a better vitamin D status [56], which could potentially serve to reduce cerebral Aβ burden according to data from animal studies [27, 28]. Moreover, a significant interaction between ApoE ε4 and 25(OH)D concentrations has been reported in relation to human memory function [57]. Therefore, the links between 25(OH)D and ApoE ε4 to govern Aβ pathology is worthy of further research.

The strengths of the current study are the relatively large sample size and the availability of PET [^18^F]-florbetapir imaging data and plasma 25(OH)D measurements, providing a more accurate value for vitamin D levels, as opposed to vitamin status assessed through dietary questionnaires. In addition, we considered several cofounders, including a possible interaction between vitamin D and ApoE ε4 status. The limitations of our study included the cross-sectional design, which precluded the examination of the relationship between plasma 25(OH)D and change in cerebral Aβ load over time. PET scans were also performed throughout the 3-year period of the MAPT; therefore the study design was not truly cross-sectional. The inclusion of the time interval between baseline 25(OH)D measurements and the PET scan as a confounder in the linear regression models probably served to mitigate this bias. Furthermore, we only had data availability for vitamin D status in later life which might perhaps not be the optimal time window for studying the associations between vitamin D and cerebral Aβ.

## Conclusions

We have shown here that circulating 25(OH)D was not associated with cerebral Aβ in older adults at risk of dementia cross-sectionally. However, a longitudinal observational study including mid-life vitamin D measurements is warranted to examine the relationship between plasma 25(OH)D and cerebral Aβ accrual over time.

## Abbreviations

25(OH)D: 25-Hydroxyvitamin D
AD: Alzheimer’s disease
ApoE: Apolipoprotein E
Aβ: Beta-amyloid
CDR: Clinical Dementia Rating
MAPT: Multidomain Alzheimer Preventive Trial
MMSE: Mini Mental State Examination
n-3 PUFA: Omega-3 polyunsaturated fatty acid
PET: Positron emission tomography
SUVR: Standard uptake value ratio
